# FocusHeuristics – expression-data-driven network optimization and disease gene prediction

**DOI:** 10.1038/srep42638

**Published:** 2017-02-16

**Authors:** Mathias Ernst, Yang Du, Gregor Warsow, Mohamed Hamed, Nicole Endlich, Karlhans Endlich, Hugo Murua Escobar, Lisa-Madeleine Sklarz, Sina Sender, Christian Junghanß, Steffen Möller, Georg Fuellen, Stephan Struckmann

**Affiliations:** 1 Institute for Biostatistics and Informatics in Medicine and Ageing Research, Rostock University Medical Center, Ernst-Heydemann-Straße 8, 18057 Rostock, Germany; 2 Annoroad Gene Technology, No. 88 Kechuang 6th Street, Beijing, 100176, China; 3 Institute of Anatomy and Cell Biology, University of Greifswald, Friedrich-Loeffler-Straße 23c, Greifswald 17487, Germany; 4 Clinic for Hematology, Oncology and Palliative Medicine, Rostock University Medical Center, 18057 Rostock, Germany

## Abstract

To identify genes contributing to disease phenotypes remains a challenge for bioinformatics. Static knowledge on biological networks is often combined with the dynamics observed in gene expression levels over disease development, to find markers for diagnostics and therapy, and also putative disease-modulatory drug targets and drugs. The basis of current methods ranges from a focus on expression-levels (Limma) to concentrating on network characteristics (PageRank, HITS/Authority Score), and both (DeMAND, Local Radiality). We present an integrative approach (the FocusHeuristics) that is thoroughly evaluated based on public expression data and molecular disease characteristics provided by DisGeNet. The FocusHeuristics combines three scores, i.e. the log fold change and another two, based on the sum and difference of log fold changes of genes/proteins linked in a network. A gene is kept when one of the scores to which it contributes is above a threshold. Our FocusHeuristics is both, a predictor for gene-disease-association and a bioinformatics method to reduce biological networks to their disease-relevant parts, by highlighting the dynamics observed in expression data. The FocusHeuristics is slightly, but significantly better than other methods by its more successful identification of disease-associated genes measured by AUC, and it delivers mechanistic explanations for its choice of genes.

Computational biologists are often confronted with molecular observations on gene/protein expression levels and are asked for guidance to link molecular processes with disease phenotypes. This is motivated by the need for diagnostic markers and for therapy monitoring and it may also yield new drugs or drug targets. Ideally, the molecular data also improves the understanding of the molecular pathology of a disease. However, the sole inspection of expression levels may be insufficient to identify disease-associated genes, since the gene(s) at the root of a disease may not change in their expression. Still, assuming that there are downstream effects, expression data mapped on a biological network may enable disease gene identification. On the other hand, network topology data, while agnostic to individual diseases, may suggest well-connected genes, which are known to be enriched in disease genes.

This paper presents an evaluation of a series of gene-disease-association predictors based on expression and network data from public resources (Gene Expression Omnibus^1^ and STRING^2^). The predictions are compared with sets of gold standard genes (from DisGeNet^3^) that have been associated (manually or by text-mining) with the respective disease phenotype. Our own approach, the *FocusHeuristics*, uses both expression levels and a network for its predictions, a concept it shares with other methods such as *DeMAND*^4^ and the *Local Radiality Score*^5^. The FocusHeuristics stands out for its dynamic interpretation of the network. It extends the ExprEssence LinkScore^6^,^7^, mapping the difference or sum of log fold changes to biological networks. While condensing the network, the heuristic retains the strongest links (describing putatively relevant gene/protein interactions).

This paper compares the above mentioned methods and further includes *Limma*^8^ as a representative of a solely expression-level based approach. On the other end, some well-known network reduction methods are added, which are based on topology measures for single genes such as the *node degree* (number of interacting partners), the *clustering coefficient* (for a given node, the number of edges between its neighbours divided by the number of all such edges possible) and the *betweenness* (relative number of occurrences on shortest paths). The former two have recently been suggested as a source of disease gene markers^9^. Finally, we compare with the HITS/Authority Score by Kleinberg^10^ and the *PageRank* method^11^,^12^. We demonstrate the competitiveness of the FocusHeuristics and discuss the different contributions of gene expression and network data to predicting gene-disease-association in general.

## Results

We aim to predict disease-associated genes represented by nodes in a network, based on the network data and on gene expression data for two conditions, “healthy” and diseased. Towards this aim, our FocusHeuristics employs three different scores using the differential expression data: the log fold change scores the difference of gene expression for the two conditions (*LFC*), the differential link score (*LS*_*d*_)^6^ scores links (that is, interactions in the network, between two genes/proteins) with different activity in the two conditions, and the interaction link score (*LS*_*i*_) scores links that are highly active in both conditions. For a detailed description of the FocusHeuristics and how it generates a ranking of genes, by their degree of disease association, see the Methods section. Gene ranking by all other methods is based on their output, using the default parameters of the respective method.

The performance of an approach towards predicting disease-associated genes can be described by a ROC curve. It plots, for all possible thresholds on the ranked gene lists, produced by the method under consideration, the fraction of hits in the reference gene set (true positive rate) against the fraction of false positives (false positive rate). (The ROC curves for all methods and all diseases are presented as a supplement to this publication, see Suppl. Figure 1). The ROC analyses were performed four times. First, from the point of view of the gold standard, they were performed on the *complete reference gene sets* as provided by DisGeNet and also on *reduced sets*, where pleiotropic genes associated with 10 diseases or more are removed. The latter analyses are biologically more meaningful, and are thus presented in the manuscript, while the former are presented in the supplement. Second, from the point of view of the methods, they were performed on *all available genes*, as well as only on the method-specific 1% *top ranking genes*, which the method considers the most relevant. Again, the latter analyses are biologically more meaningful, but we present both analyses: in the top panels of figures, the results on all available genes are shown, while in the bottom panels, the results on the top ranking genes are plotted.

**Figure 1:**
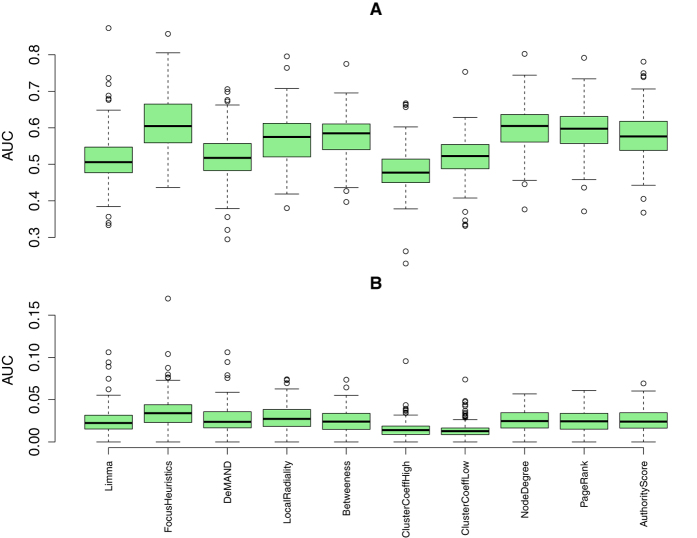
Methods scored by AUC. The box plots depict the performance of every method, scored by the area under the ROC curve (AUC), in terms of its ability to predict the non-pleiotropic gold standard disease genes provided by DisGeNet. The methods are ordered from left to right reflecting their decreasing dependency on expression data and their increasing incorporation of network data. A high AUC indicates good performance. In total, 111 data sets were used. Top panel: AUC results obtained when all genes available from the reduced reference gene set are considered. Bottom panel: AUC results when only the method-specific 1% top ranking genes from the reduced reference gene set are considered. The boxes extend from the 25^th^ to the 75^th^ percentile. The horizontal bar represents the median. Whiskers extend to points that are at most 1.5 times the interquartile range distant from the box. Measurements outside that range are plotted individually.

**Figure 2:**
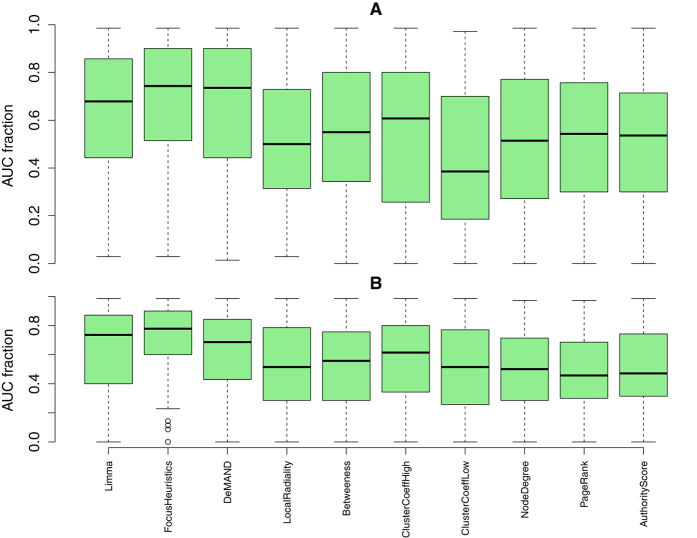
Methods compared by disease-specificity. The AUC for each disease on the matching expression data is compared with the AUC on expression data of all other diseases. In total, 111 data sets were used. In analogy to the presentation of a permutation test, the y axis indicates the fraction of diseases that trigger lower AUCs for expression data that do not match. Disease-specific methods achieve high fractions. Top panel: AUC results obtained when all genes available from the reduced reference gene set are considered. Bottom panel: AUC results when only the method-specific 1% top ranking genes from the reduced reference gene set are considered. See Fig. 1 for further explanations.

A large area under the ROC curve (AUC) indicates a well performing method–a perfectly performing method would gain a maximum true positive rate for any false positive rate, especially also for low false positive rates or even for no false positives at all, and therefore the AUC would then reach its maximum value of 1. Figure 1 presents the average AUCs across all diseases, evaluated on the reduced (but relevant) reference gene sets, and in the following we will compare methods by the median AUC they achieve. The FocusHeuristics is performing equally well as two of the topology-based methods (NodeDegree, and PageRank), if all available genes are considered (Fig. 1, top panel); it achieves the highest median if only the more biologically relevant top ranking genes are considered (Fig. 1, bottom panel). In this case, the LocalRadiality score is the second best. For the complete reference gene sets, the FocusHeuristics is still valued high for its AUC, on par with LocalRadiality and Betweenness, barely superseded by three topology based methods (NodeDegree, HITS/Authority Score and PageRank, see Suppl. Figure 2, top panel). The FocusHeuristics combines gene expression data and a network to predict genes for a single disease under investigation. When the assignment of a disease to an expression data set based on DisGeNet is changed, so that the disease is assigned to some other (control) gene expression data set, which, via DisGeNet, is associated to a different disease, then the AUC should drop. The larger the difference in AUC between the original and the control data set selection, the more disease-specific are the findings of the method. For all available genes, the top panel of Fig. 2 shows the fraction of the AUC achieved on control data that are below the AUC of the data set that matches the disease. The FocusHeuristics achieves highest ratios and hence is the approach that shows a clear tendency for drifting towards the right disease. DeMAND is the runner-up, followed by Limma, albeit Limma does not consider network information for its analysis. As expected, the network-centric approaches, which do not incorporate expression data, perform worse as by themselves these do not involve any gene expression data set. Results are very similar when the evaluation is solely based on the respective top-ranked genes, see the bottom panel of Fig. 2, except that the performance of DeMAND is slightly worse. (See Suppl. Figure 3 for the same analysis, using the complete reference gene sets.) To some degree performance differences could be caused by the unknown amount of false negatives in the respective gold standards for the various diseases. In any case, we performed an estimation of the statistical significance of the performance differences. Specifically, based on the distributions of AUCs in Fig. 1, we calculated the statistical significance of the difference in median AUC for every pair of methods (M1, M2), employing a one-sided paired Wilcoxon-Test to test whether M1 has a higher median than M2, and vice versa. We found that, despite being small, the improvement in performance of our method was statistically significant, see Supplementary Figure 10.

**Figure 3:**
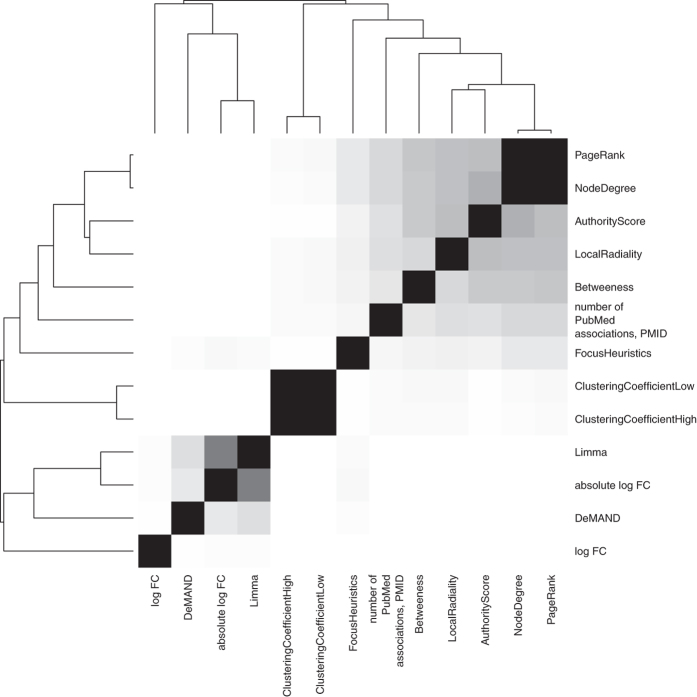
Heatmap of similarities between methods. Grey-coded similarities of two methods reflect the mean of the pairwise similarity rank correlations across all data sets. The depicted dendrogram reflects a clustering of the methods by their distance, which is directly derived from their similarity. The disease specific similarities behind this figure are given as supplementary files, see Suppl. Figure 4.

Figure 3 shows the overall distance between the predictions provided by the methods we compare. For all method pairs, the mean rank correlation between their predictions for all data sets is computed. The expression-based rankings are close to each other, and the same holds for the topology-based rankings. The hybrid methods are in the centre and the FocusHeuristics is moderately similar to both clusters of methods, since it is in the centre of the figure and shows some similarity to all topology methods, the combined method DeMAND, as well as absolute log fold change and Limma, which are expression based. The number of PubMed associations (PMID) can also be used to rank genes. This “method” is compared to the other methods to elucidate why topology-based methods work, see the Discussion below. (See Suppl. Figure 4 for the pairwise comparisons of the rankings that were summarised into distances in Fig. 3, exemplified for the disease Acute Pancreatitis. For this disease, we also provide the individual ROC curves in Suppl. Figure 1, and the AUCs for each prediction method in the context of the background distribution of AUCs in Suppl. Figure 5).

**Figure 4:**
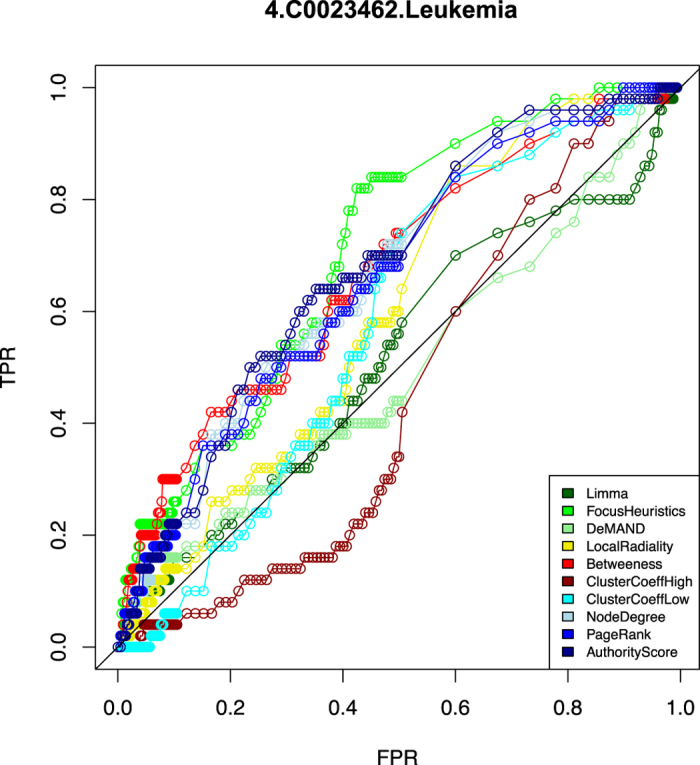
ROC curves for Acute Megakaryoblastic Leukemia (AMKL). To exemplify the concept of ROC curves, for AMKL and its corresponding gene expression data set, the ROC curves for each method are shown. The x-axis represents the specificity-related false positive rate (FPR), the y-axis sensitivity by the fraction of reference genes predicted (TPR, true positive rate). Methods are delineated by colour. The genes from the reduced reference data set (pleiotropic genes associated with 10 diseases or more have been removed, see Results section) were used. In the online supplement (http://focusheuristics.expressence.de/) respective figures are given for all 111 investigated data sets.

**Figure 5:**
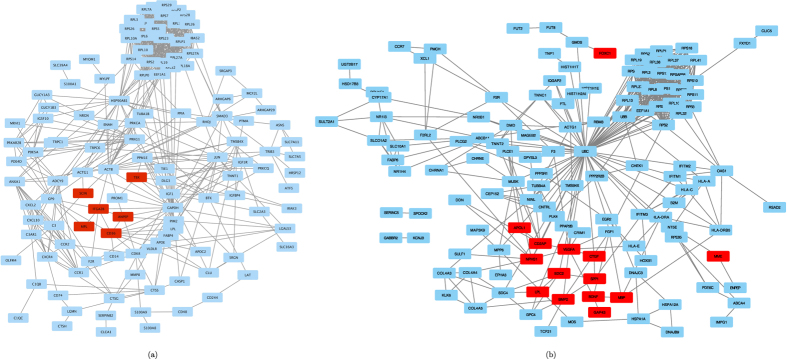
The STRING networks used by the FocusHeuristics in case of (**a**) AMKL and (**b**) diabetic nephropathy. The disease genes (see text) are marked in red.

For the specific case of acute megakaryoblastic leukemia (AMKL, also known as AML-M7), which is a rare subtype of acute leukemia and associated with negative outcome and bad prognosis, we found that our method is better than the other methods based on the area under the ROC curve, see Fig. 4. Assuming that the improved performance of our method is due to its selection of disease-relevant genes based on the links that connect them, we investigated the true positive predictions of our method, as well as the subnetwork of links between them as it was supplied to the FocusHeuristics by the underlying STRING network (Fig. 5). Here, the true positive predictions of our method are the genes predicted at a false-positive rate of 0.5 that were matching the gold-standard DisGeNet list of genes for AMKL, and they are called “AMKL genes” in the following. AMKL has not been characterized molecularly as extensively as other subtypes, but based on the overall similarity of subtypes, many aspects of these, and of AML in particular, should also be valid for AMKL.

Most notably, the AMKL genes are not scattered around in the STRING network. Instead, their subnetwork is tightly connected, as can be seen in Fig. 5a. Specifically, ITGA2B was identified as the most central AMKL gene in the subnetwork, showing the largest number of interactions with the other AMKL genes; it is one of the ITGA (integrin alpha) family members, and it is able to influence, via FAK (focal adhesion kinase), the PI3K pathway. PI3K itself is well known to act as a central hub in the pathogenesis of acute leukemia subtypes^13^. Interestingly, ITGA2B interacts with ANPEP (also known as CD13), which is a myeloid marker. This marker was found strongly expressed in AML subtypes M1-M5 indicating an important general role in of ANPEP in AML^14^. Further, targeting of CD13/ANPEP by monoclonal CD13 antibodies resulted in induction of caspase-dependent apoptosis in AML cells^15^. Additionally, the subnetwork includes the interaction of ANPEP with the TEK tyrosin kinase, via promenin1 (PROM1, also known as CD133). TEK receptor as well as ligand expression were observed in AML and CML patients and were discussed to be involved in the pathogenesis of myelo-proliferative disorders^16^. In general, TEK has many key functions in the cell, such as reorganisation of the actin cytoskeleton. Thus, the connection of TEK with ACTB via PROM1 is of special interest. ACTB (beta actin) as a housekeeping gene is essential for cell survival, thus it is not surprising that deregulations of ACTB have been described to be involved in curtailing oncogenic processes such as accelerating tumor formation, invasion and metastasis^17^. Moreover, the myeloproliferative leukemia protein MPL, that is interacting with ITGA2B, regulates the proliferation of hematopoietic stem cells and *megakaryocytes* by JAK/STAT, ERK/MEK, and PI3K/AKT pathway activation^18^. Thus, MLP could be a key for the AMKL phenotype. Finally, SCIN (scinderin) is closely connected (via ACTB) with the subnetwork of AMKL genes. The expression of this actin-severing protein was found to be important for the promotion of *megakaryoblastic* leukemia cell differentiation, maturation, and apoptosis^19^. More specifically, the lack of SCIN in megakaryoblastic leukemia cells and, consequently, the lack of proper actin dynamics, might be related to the inability of these cells to enter into differentiation and maturation pathways^19^. Thus, SCIN could be another key for the characteristic AMKL phenotype.

In case of LocalRadiality, which achieves the second-highest median if only the more biologically relevant top ranking genes are considered (Fig. 1, bottom panel), the DisGeNet gold-standard genes are AKT1, JAK2, BCL2L1, TGFB1, TFRC, AURKA and BIRC5. These are all pleiotropic genes (that is, they are listed for 10 diseases or more by DisGeNet); the FocusHeuristics highlights only two such genes (TEK, CD36), whereas the other genes (SCIN, ITGA2B, ANPEP and MPL) can be considered non-pleiotropic and more disease-specific.

We investigated a second specific case, i.e. diabetic nephropathy (DN). DN is the most common form of chronic kidney disease. However, the pathogenesis of DN remains incompletely understood and treatment options are limited^20^,^21^,^22^. In analogy to AMKL, we investigated the true positive predictions of our method, the “DN genes”, as well as the subnetwork of links between them (Fig. 5b). Notably, 12 of the 14 identified DN genes form a subnetwork. This subnetwork contains three genes, NPHS1 (nephrin), CD2AP and VEGFA, that are highly expressed in podocytes and that are essential for the proper function of the glomerular filtration barrier^23^,^24^,^25^. The role of the podocyte and its paracrine signaling to the glomerular endothelium is well established in the pathogenesis of DN^20^,^26^. In addition, the FocusHeuristics identified two matricellular proteins CTGF and SPP1 (osteopontin). Both proteins have been shown to be involved in DN. CTGF, originating from systemic and renal sources, is elevated in the urine of patients with DN^27^. Osteopontin has been described as a lead classifier for kidney failure-associated morphological alterations in the kidney^28^. As the early stage of DN is characterized by glomerular hyperfiltration, it is interesting to note that osteopontin induced by mechanical stress protects podocytes via binding to *α*_*V*_-integrins^29^,^30^.

## Discussion

The FocusHeuristics described in this paper selects genes, based on the links that connect them, of a biological network that best reflect observations in a gene expression experiment. It has the dual application to constrain candidate lists for disease-associated genes and to determine a disease-specific subnetwork still considering both, topological and expression-based information (see Fig. 3). The latter renders the associated molecular pathways better accessible to both the human observer and to computationally demanding algorithms. Our method contributes a new competitive approach (see Fig. 1) that is dissimilar to other solutions in the field (see Fig. 3). It shows the largest advantage in its specificity for the disease under investigation (Fig. 2). With it, disease-associated genes are found at lower costs introducing fewer false positives than by the interpretation of expression levels alone.

DisGeNet lists a high number of pleiotropic (unspecific) genes annotated for 10 diseases or more. Considering such disease-unspecific a priori knowledge for the evaluation, a method preferably predicting disease-specific genes may not achieve high AUC scores. As a consequence, to allow the evaluation to reflect the capability of the methods to identify disease-specific genes, all analyses were done with pleiotropic genes masked out. As expected, on the complete gene reference set, the topology-focused methods are performing well, but especially for the non-pleiotropic genes they fall behind (as indicated in Fig. 2). Pleiotropic genes tend to have a higher connectivity, i.e. they are more prone to be hub genes (see Suppl. Figure 6). Methods that use only connectivity are disease-agnostic. However, topology-based methods indeed work quite well to predict disease-gene-associations (including unspecific disease-associated genes) as shown in Fig. 1. Also, pleiotropic genes are likely to be also well-studied genes and thus they are over-represented in the literature, featuring higher node degrees. Moreover, Fig. 3 shows that the ranking of genes by their sheer number of Pubmed citations (PMID) clusters together with the topology based methods.

This paper presents a robust method to condense a biological network by highlighting links, and the genes they are connecting, based on gene expression data. It is a hybrid approach, combining information from network topology and expression data, which, especially for non-pleiotropic genes, is better than the state of the art if compared employing AUC (see Figs 1 and 2). Especially the exclusively expression- or topology-centric methods are worse than the FocusHeuristics in particular and they are worse than combined approaches in general. Since the FocusHeuristics reduces a network to genes and links that are either changed or highly active in the observed conditions, it can be used as a module in a network-based analysis pipeline to add this context dependency. The modular nature of the FocusHeuristics facilitates its integration in a wide array of applications, it is available as an R package from http://focusheuristics.expressence.de. The FocusHeuristics can be combined with other methods that were presented in the literature, e.g. the evolutionary age of a gene^31^ or the intergenomic consensus QTL^32^, which may all be applied as additional filters or aggregators. Also, the resulting gene ranking may be subject of a functional enrichment analysis. Finally, the condensed network provided by the FocusHeuristics may serve as input for other tools facilitating integration into larger workflows, e.g. with netGSA^33^,^34^ to work on all-to-all shortest paths.

## Methods

### FocusHeuristics

Biological functional networks, e.g. STRING^2^, Reactome^35^ or the Selventa Knowledge base^36^, model relations between genes or proteins (such as gene regulation or protein interaction) as graphs. The proposed method condenses biological interaction/regulation networks based on differential gene expression. The FocusHeuristics first computes three scores: The log fold change (LFC), i.e. the log-transformed difference of gene expression for two conditions, the differential link score (*LS*_*d*_)^6^, and the interaction link score (*LS*_*i*_). In more detail, the two link scores assess graph edges, i.e. gene/protein links, by gene expression. The differential link score is the sum (for activation and un-specified links) or the difference (for inhibitions) of the *LFCs* of the connected genes/nodes. The interaction link score is introduced to express the activity of a link in the network for both conditions. To compute the interaction link score, the (log-)expression levels of the connected genes are summed up for both conditions; the minimum of these two sums is the interaction link score *LS*_*i*_. The FocusHeuristics generates a network by keeping all edges and nodes from the reference network that pass at least one of the thresholds for the above described three scores. These thresholds are parameters of the algorithm. In our analyses, we run the FocusHeuristics with decreasing thresholds, giving rise to ROC curves (see below). Formal definitions of the scores are as follows:


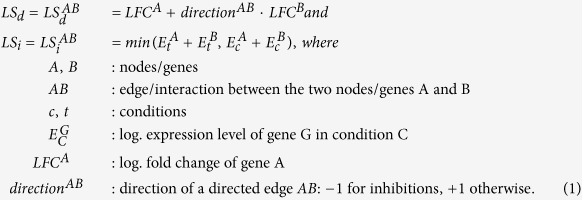


### Evaluation

All methods participating in the comparison yield a ranking of input genes reflecting the predicted disease association of the genes. The output of the FocusHeuristics is a condensed network. We derive a ranked list of genes by applying the FocusHeuristics as follows. The size of the condensed network depends on thresholds for the three scores described above. By decreasing these thresholds, different network sizes are generated, and the gene(s) occurring in the smallest network (reduced to size 1) are the most important, the gene(s) occurring in the second smallest network are the next important ones, and so on. All methods are applied to the genes that are present in both the expression data and the network. The only exception is Limma, which is run on the whole expression data set, but its output is subsequently filtered for genes that are in the network.

Sirota *et al*.^37^ provide a list of disease annotations for Gene Expression Omnibus^1^ data sets. Based on matching this list with the diseases considered in DisGeNet, the respective expression data set(s) can be used for evaluation. Each data set had to comprise “healthy” and disease samples, and each class of samples had to provide at least 3 replicates. The data is post-processed to uniformly provide log fold changes and annotations with the corresponding disease, following the *Concept Unique Identifier* (CUI). The complete set of gene-disease associations was downloaded in version 3.0 of DisGeNet and used without further filtering. DisGeNet identifies the diseases by their CUI, allowing for seamless matching of a disease gold-standard gene set to an expression data set describing the very same disease. Reference gene sets for 70 different diseases are thus identified that match to 111 gene expression data sets, allowing for multiple gene expression data sets to be assigned to a single disease. Suppl. Data 2 lists all assignments of diseases in DisGeNet to entries in the Gene Expression Omnibus. (Several databases have been developed to store associations between genes and diseases such as GeneCards^38^,^39^, CTD^40^, OMIM^41^ and the NHGRI-EBI GWAS catalog^42^, and we found that DisGeNET is the most comprehensive knowledgebase with CUIs).

The emphasis of this investigation is on disease-specific genes, reflecting biological relevance, which is increased in two ways. Firstly, the complete reference gene sets a are used, that is, the full set of genes in the network, as well as reduced sets, consisting of the non-pleiotropic genes (that is, the 714 of 8899 DisGeNet genes associated with 10 diseases or more were masked from the evaluation, see Suppl. Data 1). Secondly, the case of reduced sets is biologically more meaningful, so it is presented in Fig. 1, whereas the case of complete reference gene sets is presented in the supplement. Moreover, the case of complete reference gene sets, as well as the case of reduced sets, each gave rise to two variants of the analysis, so that overall, four analyses were conducted and four sets of results are presented. The first variant (top panels of figures), presents results based on all available genes. The second variant (bottom panels) imposes a further restriction, that is, only the method-specific 1% top ranking genes are considered here.

The evaluation is performed with the network provided by the STRING database^43^ version 10, with duplicated edges and self loops removed. All links are filtered for a combined confidence score above 0.500, see Suppl. Fig. 7 for a motivation of this threshold. ENSEMBL protein IDs are mapped to HUGO gene symbols. Figure legends show the names of methods with the exception of the Clustering Coefficient (ClusterCoeff). The latter comes with two variants, indicating that a high or a low value of the coefficient was considered superior for the ranking.

Most computations were performed on a local Ubuntu Linux machine^44^ running R 3.2.2^45^. The packages for *Limma*^8^,^46^ and *DeMAND*^4^ are provided by BioConductor^47^. The *LocalRadiality Score*^5^ is extracted from code from the respective online supplement. Network operations are performed using the *igraph* package^48^ within BioConductor. This package also provides the graph-theoretical measures^49^ and the implementations of *HITS/Autority Score* and *PageRank*. The method *PMID* refers to the number of PubMed associations for a gene as presented by the NCBI gene2pubmed table^50^,^51^ from which a ranking is derived. For details about the contributing packages and versions please refer to the supplement.

## Additional Information

**How to cite this article:** Ernst, M. *et al*. FocusHeuristics – expression-data-driven network optimization and disease gene prediction. *Sci. Rep.* **7**, 42638; doi: 10.1038/srep42638 (2017).

**Publisher's note:** Springer Nature remains neutral with regard to jurisdictional claims in published maps and institutional affiliations.

## Supplementary Material

Supplementary Information

Supplementary Table

Supplementary Data

Supplementary Rsession Info
