# Supplementary material for: FocusHeuristics – expression-data-driven network optimization and disease gene prediction
Source: Sci Rep. 2017 Feb 16;7:42638. doi: 10.1038/srep42638 (PMC5311990; doi:10.1038/srep42638)
Supplement: Supplementary Information [file srep42638-s1.pdf]

# FocusHeuristics – expression-data-driven network optimisation and disease gene prediction

**Mathias Ernst[1], Yang Du[1,2], Gregor Warsow[1,#], Mohamed Hamed[1], Nicole Endlich[3], Karlhans Endlich[3], Hugo Murua Escobar[4], Lisa-Madeleine Sklarz[4], Sina Sender[4], Christian Junghanß[4], Steffen Möller[1], Georg Fuellen[1,*], and Stephan Struckmann[1,*]**

[1]Institute for Biostatistics and Informatics in Medicine and Ageing Research, Rostock University Medical Center, Ernst-Heydemann-Straße 8, 18057 Rostock, Germany
[2]Annoroad Gene Technology, No. 88 Kechuang 6th Street, Beijing, 100176, China
[3]Institute of Anatomy and Cell Biology, University of Greifswald, Friedrich-Loeffler-Straße 23c, Greifswald 17487, Germany
[4]Clinic for Hematology, Oncology and Palliative Medicine, Rostock University Medical Center, 18057 Rostock, Germany,

[#]Present address: Division of Theoretical Bioinformatics (B080), German Cancer Research Center (DKFZ), Im Neuenheimer Feld 280, D-69120 Heidelberg, Germany
[*]Correspondence to fuellen@uni-rostock.de

## SUPPLEMENT

## Organisation of the supplement

This supplement comprises Supplementary Figures 1-12, and Supplementary Data 1-3.

**Suppl. Figure 1**

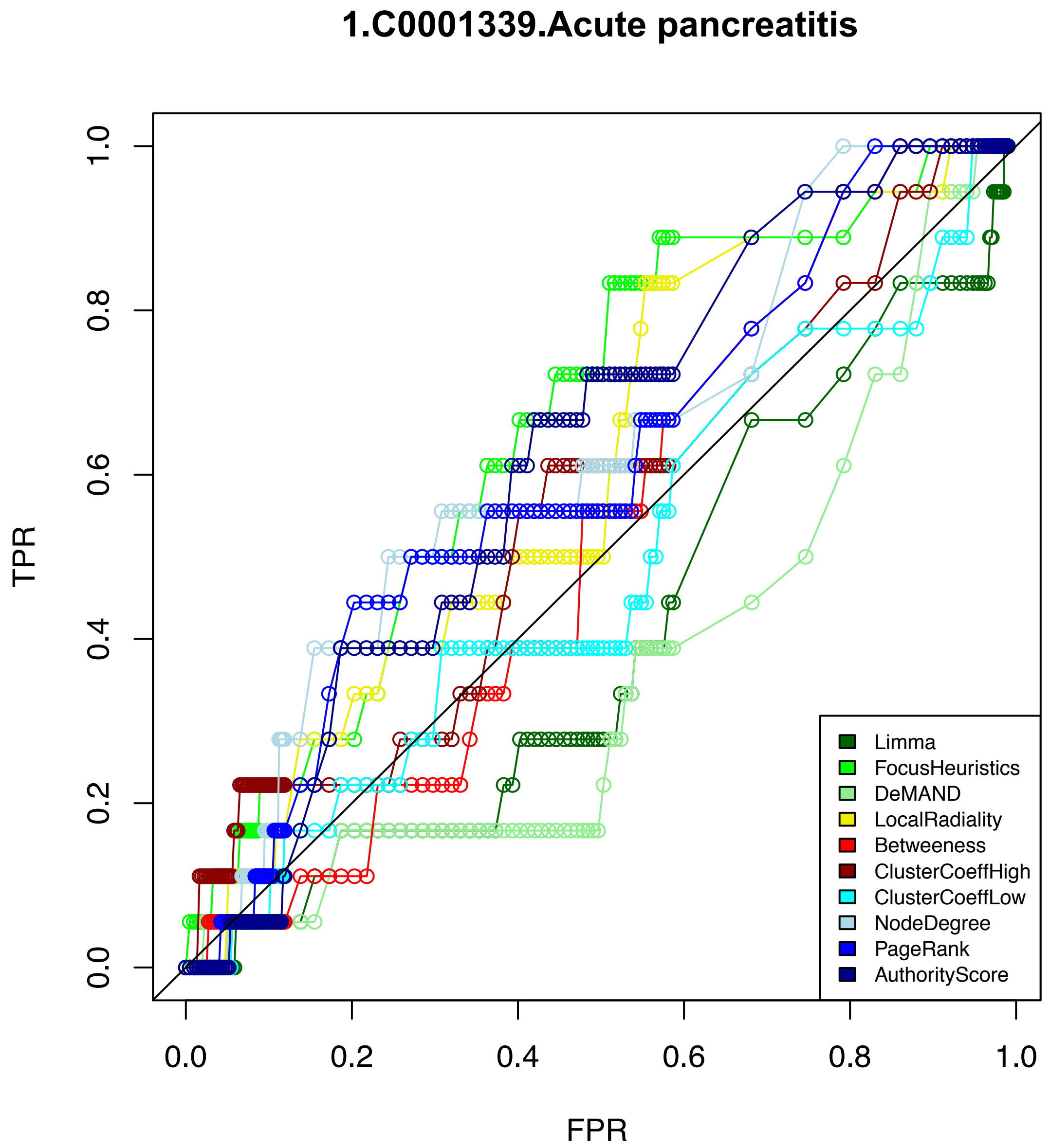


ROC curves for Acute Pancreatitis (reduced gene set). To exemplify the concept of ROC curves, for Acute Pancreatitis and one of its corresponding gene expression data sets, the ROC curves for each method are shown. The x-axis represents the specificity-related false positive rate (FPR), the y-axis sensitivity by the fraction of reference genes predicted (TPR, true positive rate). Methods are delineated by colour. In the online supplement (http://focusheuristics.expressence.de/) respective figures (”ROC plots”) are given for all 111 investigated disease data sets.

## Suppl. Figure 2

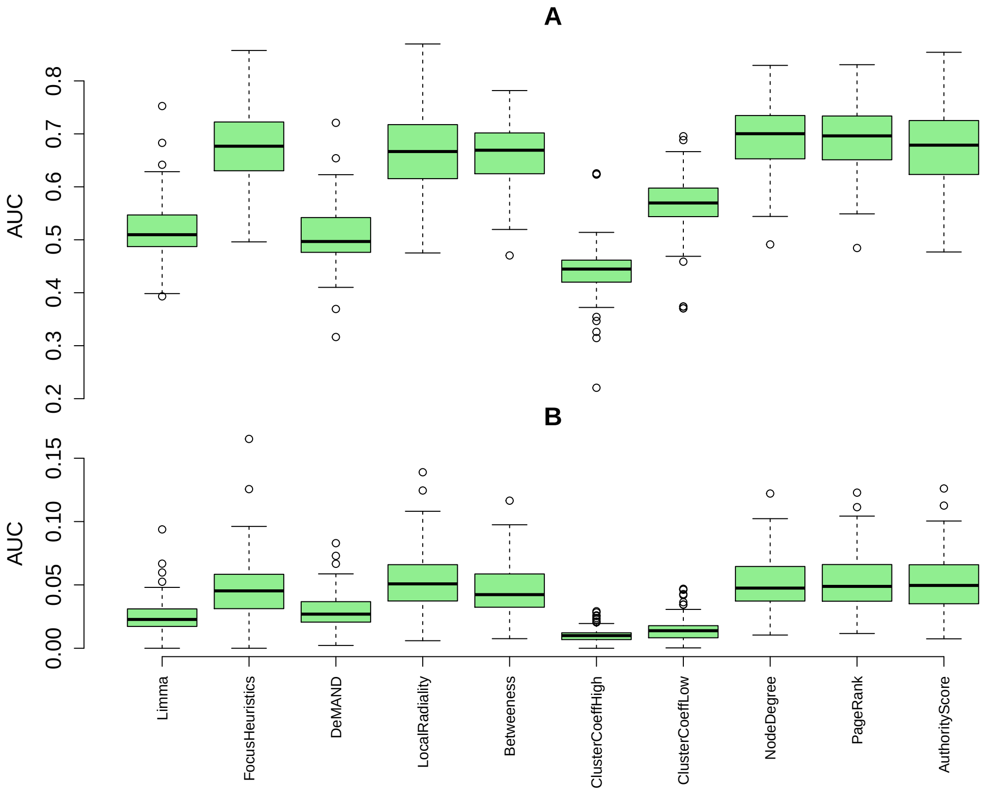


Methods scored by AUC, determined on the full reference gene set, i.e. without removing pleiotropic disease genes that are annotated with more than 10 diseases in DisGeNet. Analogous to Figure 1 of the main text, the box plots depict the performance of every method, scored by the area under the ROC curve (AUC). Top panel: results obtained when all available genes are considered. Bottom panel: results when only the 1% top ranking genes are considered. See Figure 1 of the main text for further explanations.

## Suppl. Figure 3

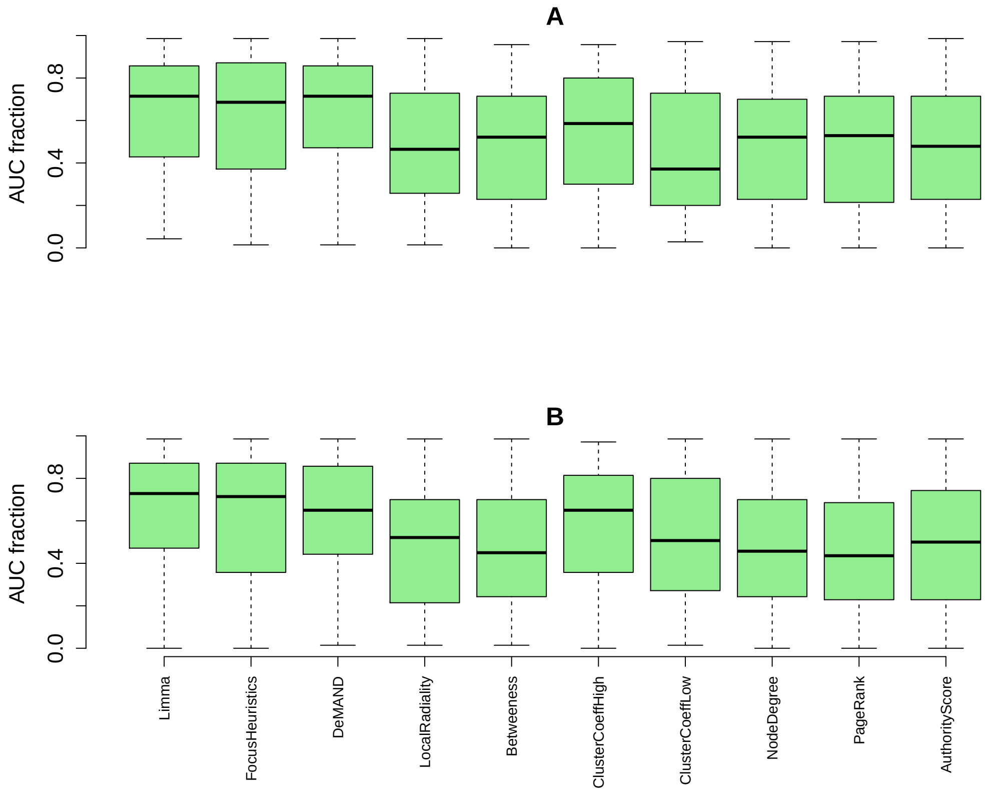


Methods compared by disease-specificity, determined on the full reference gene set. Analogous to Figure 2 of the main text, the AUC of each disease on the matching expression data is compared with the AUC on expression data of another disease. See Figure 2 of the main text for further explanations, also regarding the difference between the top and the bottom panel.

## Suppl. Figure 4

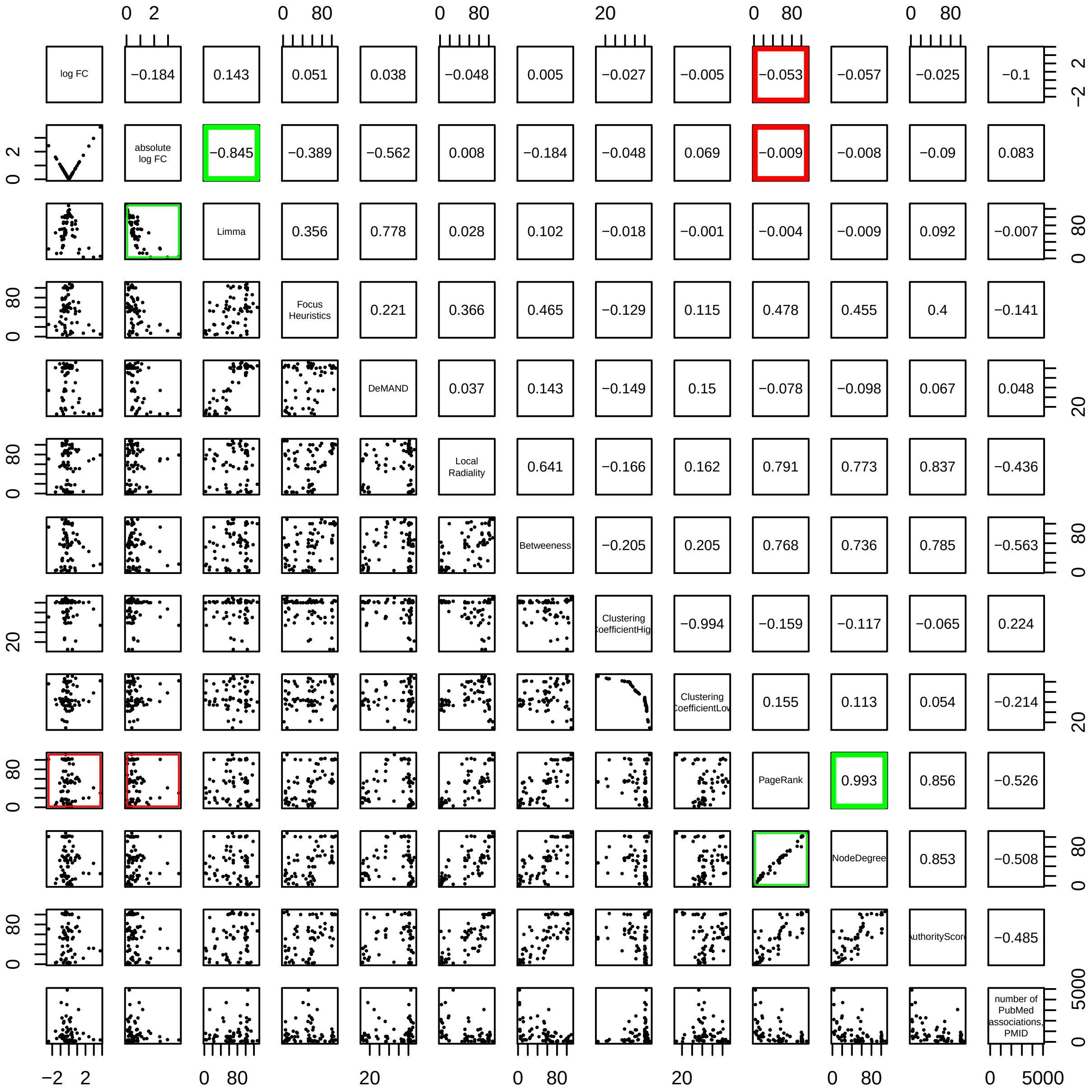


Pairwise comparison of rankings, exemplified for Acute Pancreatitis. In Figure 3 of the main text, method similarities are presented. For each disease, these are based on a comparison of the rankings provided by the individual methods. We determine the quantile at which each gene associated with Acute Pancreatitis appears for the first time in the result list for each of the given methods; a good method is expected to feature many disease-associated genes in the low quantile part (these genes are thus picked up early). In the lower triangle of the scatterplot matrix we plot those values for the methods against each other. Some additional prediction methods are also included, namely the log fold change, the absolute log fold change, the number of direct neighbours in the network (degree) and the number of scientific publications that are annotated to that gene in *gene2pubmed* (PubMed associations, PMID). The upper triangle gives the pairwise Spearman correlations: the cell $ji$ shows the numeric Spearman rank correlation corresponding to the scatterplot in cell $ij$ ($i$ and $j$ give the row $i$ and the column $j$ of a cell). Note that the methods are ordered according to the extent to which they rely on gene expression data (from Limma to PageRank); accordingly the methods are preceded by the fold change measures and followed by degree and number of PubMed associations. Four examples, two highly correlated methods (Limma and the absolute log fold change as well as PageRank and the NodeDegree) and two poorly correlated methods (the absolute log fold change and PageRank as well as the log fold change itself and PageRank), are highlighted in red and green respectively. For the highly correlated methods, the dots are close to the diagonal while for the uncorrelated methods, they are at random positions. In the online supplement (http://focusheuristics.expressence.de/) respective figures ("Scatter Matrix plots") are given for all 111 investigated disease datasets.

**Suppl. Figure 5**

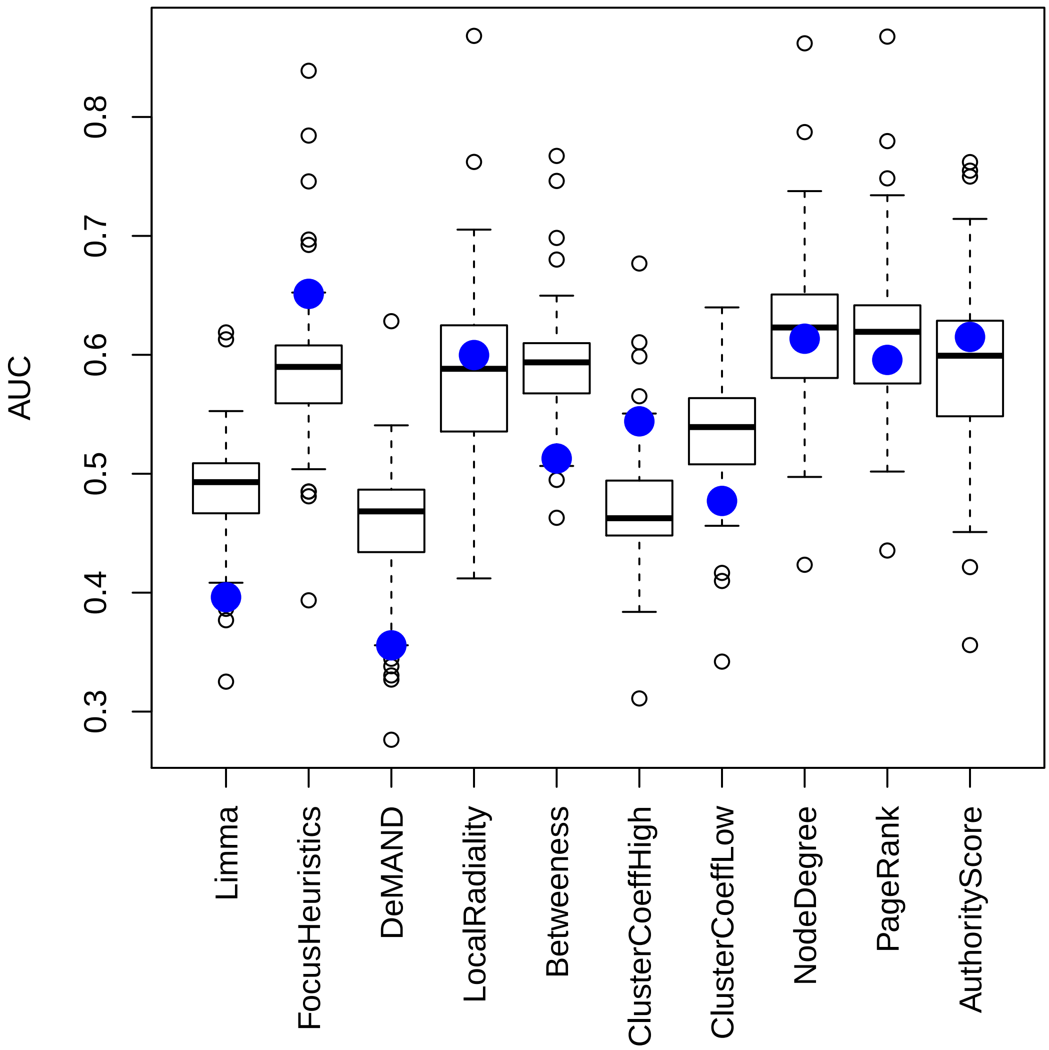


Comparison of the methods by their respective Areas Under the (ROC)-Curves. Exemplified by Acute Pancreatitis and one corresponding gene expression data set, the AUCs for each method are compared. The blue dots represent the AUC values for each method, the boxes give the background calculated by using the reference data sets for all diseases except Acute Pancreatitis (C0001339). In the online supplement (http://focusheuristics.expressence.de/) respective figures (”AUC Box plots”) are given for all 111 investigated disease data sets.

## Suppl. Figure 6

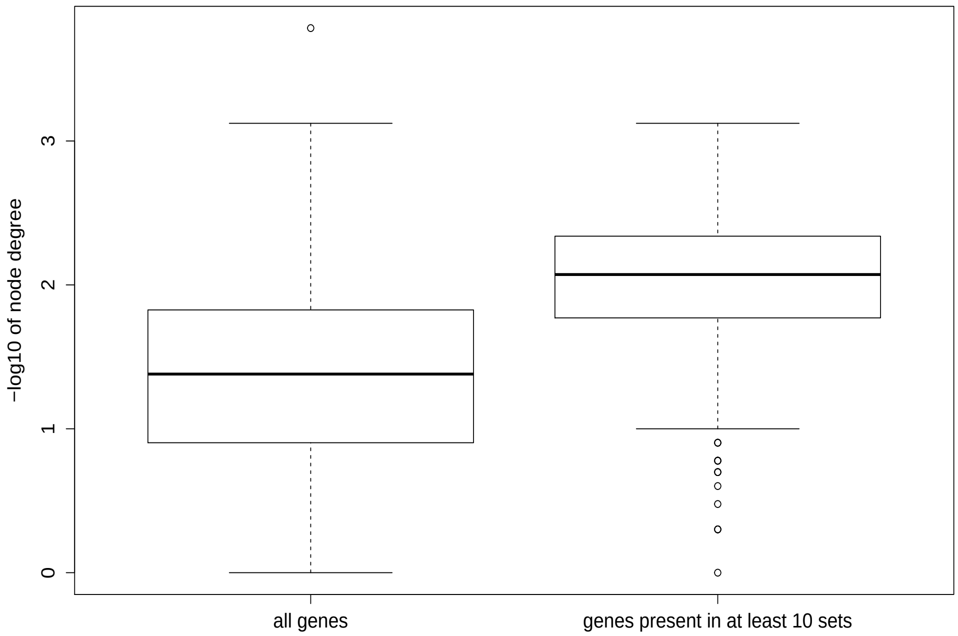


Pleiotropic genes (boxplot on the right) are more likely to be hub genes in the network.

## Suppl. Figure 7

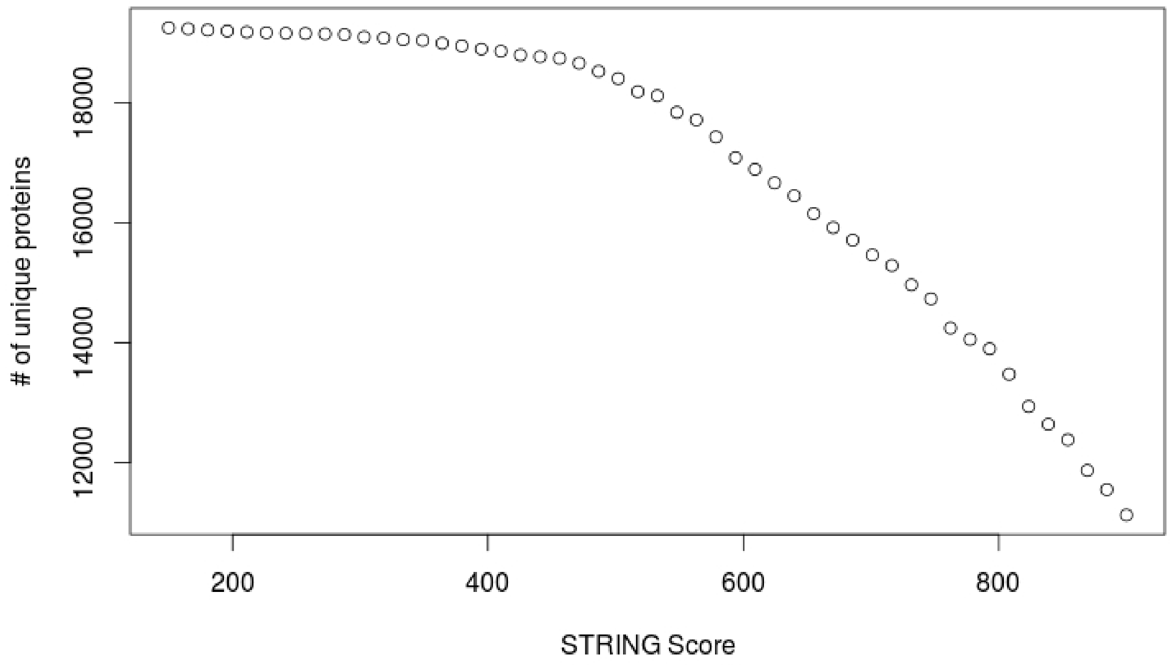


Number of nodes (genes/proteins) of a score-filtered excerpt of the human STRING 10 network displayed as a function of the STRING combined score. At a value of 500, protein coverage starts declining, and therefore 500 was chosen as a threshold for the network underlying the analyses in the manuscript.

## Suppl. Figure 8

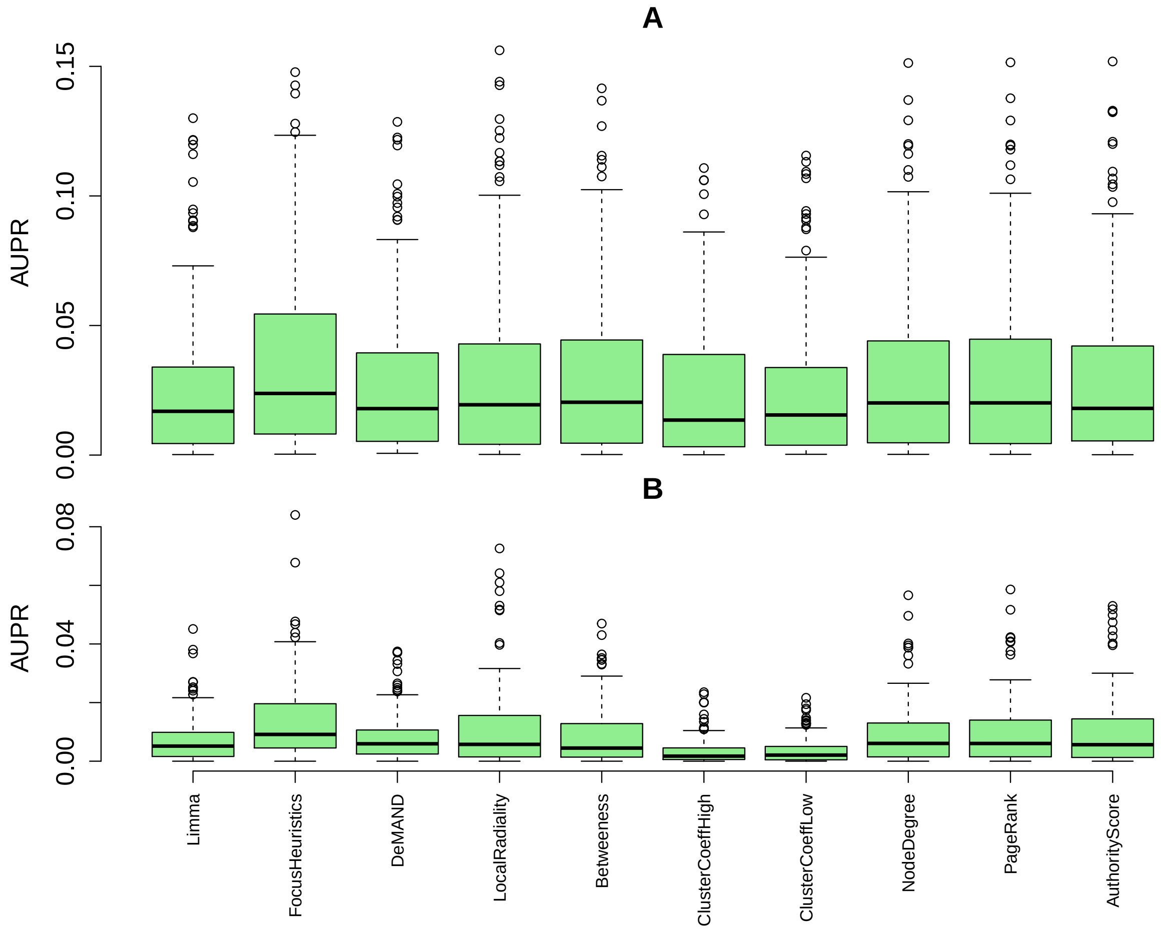


Methods scored by AUC, using the Precision-Recall curve. All other parameters are the same as in Figure 1 of the main text; see the legend of Figure 1 for further explanations

## Suppl. Figure 9

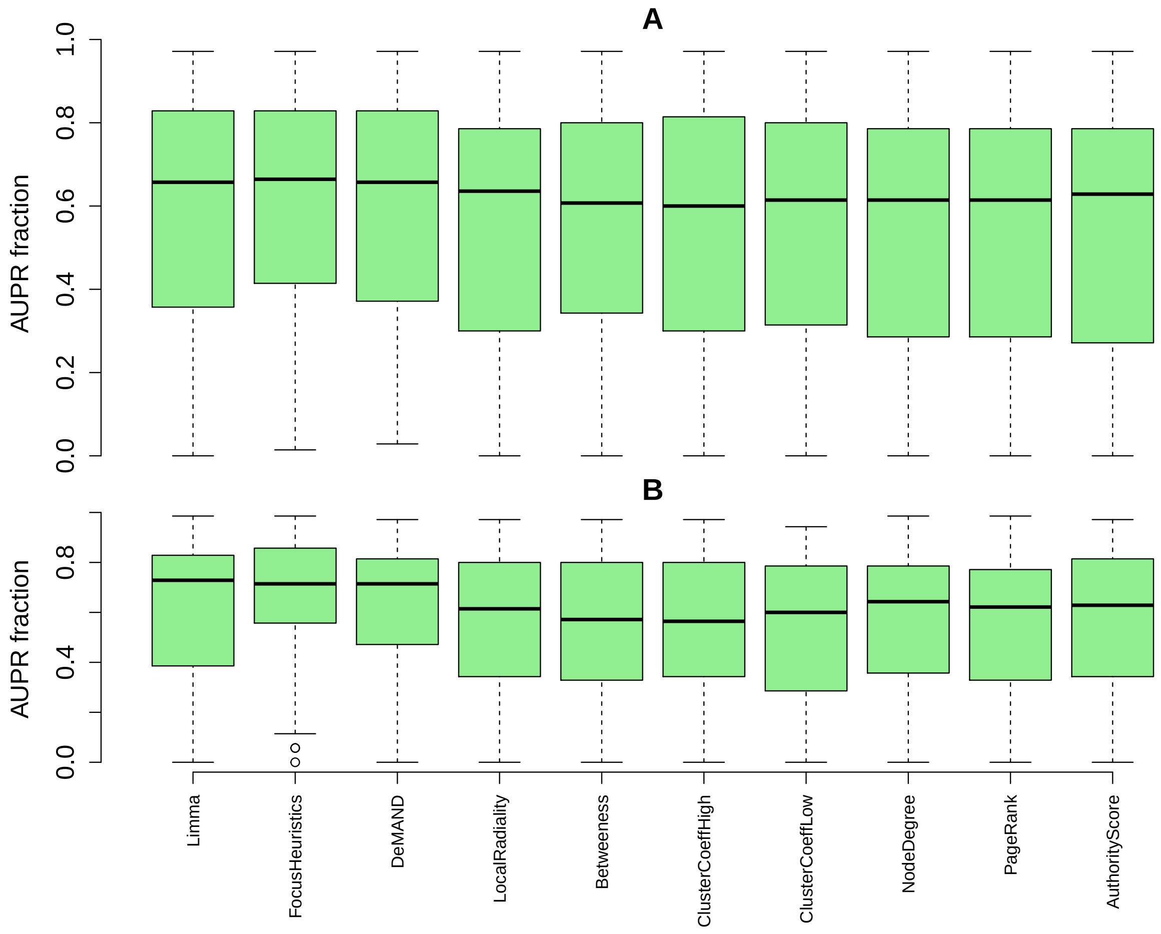


Methods compared by disease-specificity, using the Precision-Recall curve. All other parameters are the same as in Figure 2 of the main text; see the legend of Figure 2 for further explanations.

## Suppl. Figure 10

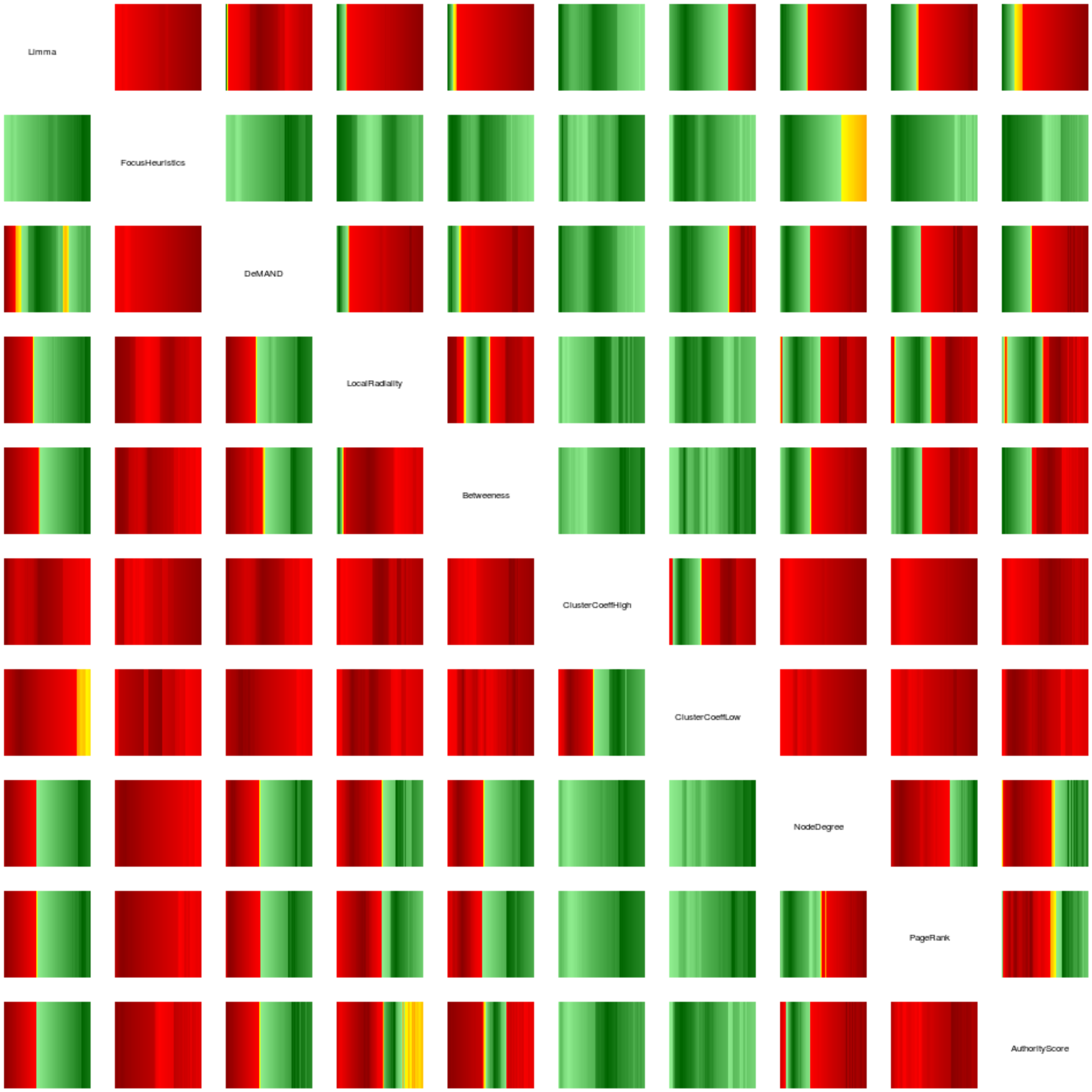


Pairwise method comparison, describing significance of performance differences. The diagonal designates the methods that are part of the comparison; below and above the diagonal, the significance of the performance differences for this comparison (”row” method versus ”column” method) is visualized by a spectrum. The x axis of the spectrum represents the number of genes selected from the rankings generated by the methods, starting with the single gene scored best by the first method, and the single gene scored best by the second method; each gene will be checked with the gold standard, and the number of genes taken from the rankings is then extended gradually. The color corresponds to the p-value obtained by a one-sided paired Wilcoxon test for a significantly higher median AUC of the ”row” method versus the ”column” method, when the number of genes given by the x-coordinate is selected from the top of the rankings of the methods to be compared. Shades of green indicate $p < 0.05$, shades of yellow indicate $0.05 < p < 0.1$, shades of red indicate $p > 0.1$. For example, the second column demonstrates that no other method is significantly better than the FocusHeuristics for almost all numbers of genes. In turn, the second row demonstrates that the FocusHeuristics is significantly better than the other methods except for the NodeDegree method in case of larger gene lists (on average, if more than 7397 genes are selected). Significance tests were based on 111 disease data sets.

## Suppl. Figure 11

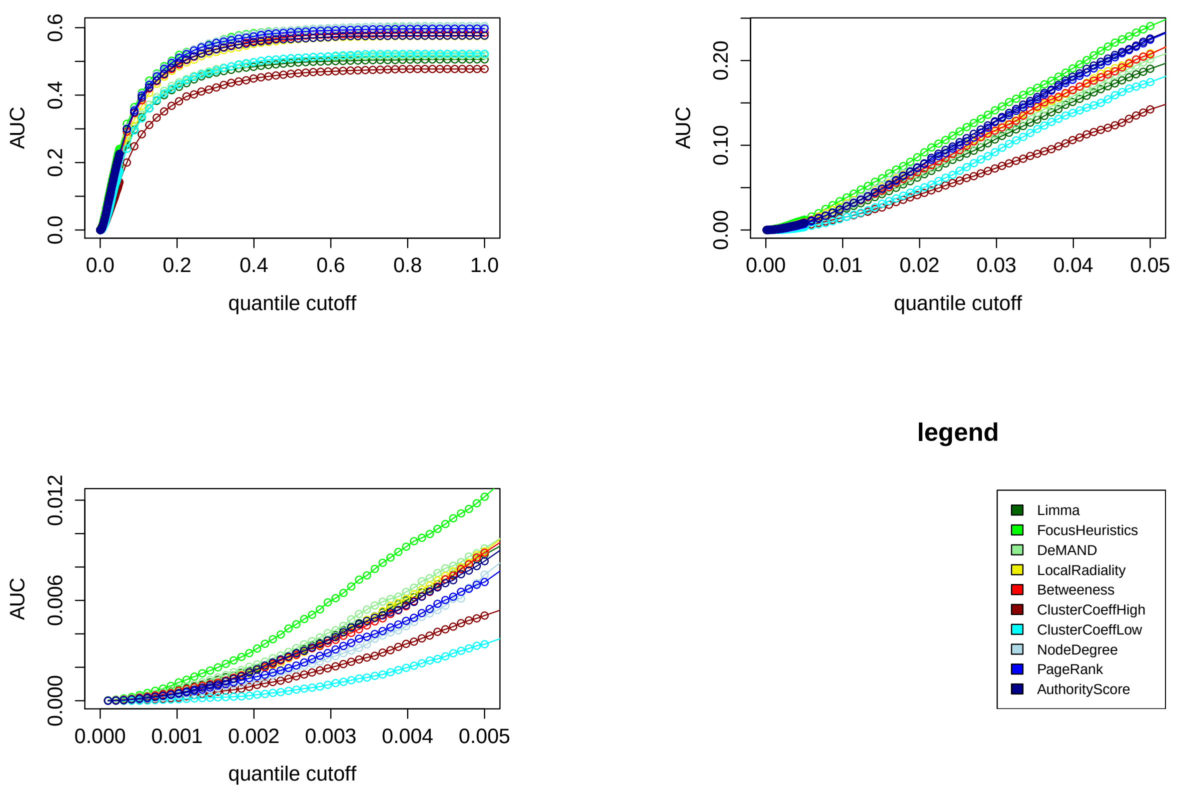


Methods scored by AUC as in Figure 1, but instead of boxplots, AUC scores are plotted as a function of the quantile cutoffs for the top-ranking genes. A: Plot considering all quantile cutoffs. B,C: Two-step zoom into the bottom-left corner of A. D: Plot legend, assigning methods to colors. See the legend for Figure 1 for further details.

## Suppl. Figure 12

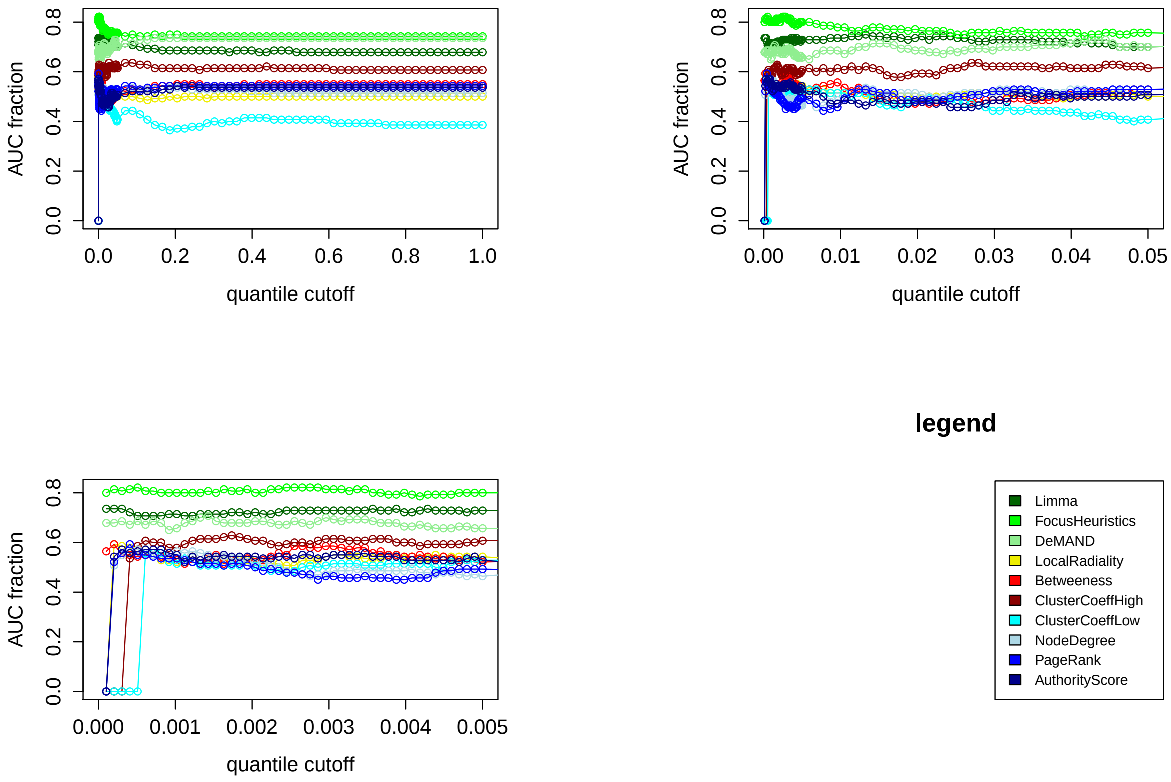


Methods compared by disease-specificity as in Figure 2, but instead of boxplots, AUC scores are plotted as a function of the quantile cutoffs for the top-ranking genes. A: Plot considering all quantile cutoffs. B,C: Two-step zoom into the bottom-left corner of A. D: Plot legend, assigning methods to colors. See the legend for Figure 2 for further details.

## Suppl. File 1

Suppl. file HubGeneTable.csv – Pleiotropic genes, i.e. genes annotated for more than 10 diseases by DisGeNet. The first column (gene) lists gene identifiers, the second the respective node degree in the STRING network excerpt that was used. The last column (num.of.annotations) contains the number of diseases each gene is annotated with by DisGeNet. The file is also available from http://focusheuristics.expressence.de/.

## Suppl. File 2

Suppl. file DataOverview.csv – The table shows the assignment of diseases in DisGeNet (column B) to entries in Gene Expression Omnibus (column I), using the CUI (columns C and J). The chips vary in the number of genes represented (column D). The number of "healthy" and disease samples are given in column E and F; the number of disease-associated genes (as taken from DisGeNet) and the size of their non-pleiotropic subset are given in column G and H. The file is also available from http://focusheuristics.expressence.de/.

## Suppl. File 3

The R package FocusHeuristics is available from http://focusheuristics.expressence.de/.

## Suppl. File 4

Suppl. File ressioninfo.txt – Version numbers of all R packages used, available from http://focusheuristics.expressence.de/.
